# Book and Pamphlet Notices

**Published:** 1859-03

**Authors:** 


### BOOK AND PAMPHLET NOTICES.

A TREATISE ON FRACTURES. By J. F. MALGAIGNE, Chirurgien de l'Hopital Saint Louis, Chevalier de la Legion d'Honneur et du Merite Militaire de Pologne, Membre de l'Academie Imperiale de Medicine. With one hundred and six Illustrations. Translated from the French. With notes and additions, by John H. Packard, M.D. Philadelphia: Lippencott & Co. 1859.

The distinguished and indefatigable Malgaigne could hardly give the profession an ordinary work on surgery. His profound learning, almost unlimited experience and tireless industry, have made him one of the greatest lights in the science of surgery, and spread his name all over the whole civilized world as such, and the profession would be disappointed if this treatise was not an excellent one.

His book will not only sustain his already well earned reputation, but very greatly advance it; indeed it is a monument that must last as a perpetual mark of honor to his memory. Dr. Packard has executed a difficult task of translating and anglicising it in an admirable manner, and deserves, as he no doubt will receive, the thanks of his medical brethren.

No man who practices surgery can so easily and perfectly accomplish himself in the mechanical surgery of the bones as becoming thoroughly acquainted with the teachings of M. Malgaigne through his treatise on fractures. It is for sale at S. C. Griggs & Co., 39 and 41 Lake street. Pages 683.

MIND AND MATTER; or, Physiological Inquiries, in a Series of Essays, intended to Illustrate the Mutual Relations of the Physiological Organization and the Mental Faculties. By Sir BENJAMIN BRODIE, Bart., D.C.L., Vice-President of the Royal Society. With additional notes by an American editor. New York: S. S. & W. Wood, 389 Broadway. 1858. For sale by S. C. Griggs & Co., 39 and 41 Lake street.

In this little semi-professional gem, Sir Benjamin shines with the lustre of a true philosopher as he is. One can hardly tell which to admire most, the science, deep philosophy and literary beauty of the work, or the graceful modesty with which the author continues to clothe his authority. In his broad and comprehensive view of the connection of mind and matter, he sees in the brain, the organ of the mind, the medium through which mental phenomena are manifested, and, like Rev. Doctor

Butler, is of opinion that in animals generally it performs the same office, that in proportion to the perfection of its organic size and structure will the perfection of mental manifestations be. No book we have ever had the pleasure of perusing is calculated to afford a more pleasant enjoyment than this. The mind is carried through difficult philosophical problems with such gentle and even progression that the reader is not aware of having passed over some of the most rugged physiological abstracts, until he is emerging from what may have seemed before a mass of confusion, with clear perceptions of one of the most sublime subjects. The theme is worthy of such a pen, and the writer has done honor to the theme. The weight of the doctrine and arguments in these treatises on mind and matter must be felt in future labors in the same direction. Pages 260.

The Uremic Convulsions of Pregnancy, Parturition and Childbed. By Dr. Carl R. Braun, Professor of Midwifery, Vienna. Translated from the German. With notes by S. Mathews Duncan, F.R.C.P.E., Lecturer on Midwifery, etc. etc. New York: Samuel S. and William Wood, 389 Broadway. 1858. For sale at S. C. Griggs & Co., 39 and 41 Lake street. Pages 182.

The translator informs us that this is a translation of a single chapter of Dr. Braun's new text book of midwifery. Dr. Braun has brought to the task which he has so thoroughly performed, the erudition, science, patience of research, and untiring perseverance which characterise our German professional brethren. He thinks *eclampsia puerperitis* is a consequence of *nephritis diffusa sen albuminosa* arising from pressure of the gravid uterus upon the veins. Thus connected, nephritis-toxemia, or uremia and eclampsia. The urine is generally acid in reaction, as shown by the proper tests, and when boiled or mixed with nitric acid, throws down a precipitate of albumen; fibrin cylinders are quite abundantly observed through the microscope, and occasionally blood corpuscles; urea is much diminished, and sometimes wanting in this secretion. Often at the time of the convulsions, there is a partial or complete suppression and tenderness and pain around the loins. Pressure over the kidneys firmly made on strong percussion causes the patient to wince even when quite stupid. So far as morbid anatomy is concerned, there seems to be no peculiar lesions of the nervous centres, except in apopletic cases, which are rare compared to the whole number. Nor will the appearances in any of the other organs account for the symptoms besides the kidneys. The kidneys are almost always found more or less hyperæmic, and often in a state of actual inflammation, "acute Bright's disease" in one of the three stages, congestion, effusion, or degeneration. To sum up: according to Dr. Braun, the uterus presses upon the veins, retards the flow through them, dams the blood back upon the kidneys, induces congestion, prevents the secretion of urea, and other excrementitious substances, causes them to be retained in the blood, thus induces toxemia, and presses out the albumen from the serum of the blood through the delicate walls of the capillary vessels. The thin watery condition of the blood permits the effusion of serum into the areolar tissue of the extremities, and sometimes of the lungs. The retained urea is probably converted by decomposition in the blood into carbonate of ammonia, which is highly poisonous, and, circulating through the nervous centres, enhance their excitability to such an extent, as to give origin to convulsions, upon the application of slight causes. One author attributes the great mortality of the children born during puerperal convulsions to the carbonate of ammonia circulating in the blood of the mother getting into the vessels of the fœtus. We are disposed to attribute much more influence to congestion of the brain produced by pressure upon the aorta, and consequent daming back the blood upon the head and upper extremities, in causing convulsions, than the Dr. Braun cerebral hyperemic in connection with uremia, and perhaps often alone. The fits cease in 37 per cent., become measles in 31 per cent., and continue with unabated severity in only 32 per cent. of the cases that occur before delivery of the child. They seem to induce premature labor in 25 per cent. Dr. Braun also thinks that sometimes the health of the child is affected by nursing the milk of a mother who has suffered from eclampsia. About 30 per cent. of cases of puerperal convulsions terminate fatally.

The prophylactic treatment consists first in measures calculated to keep the tubuli as clean of fibrinous obstructions as possible by copious diluents and gentle diuretics, and to neutralise the carb. of ammonia in the circulation by the use of benzoic acid, lemon juice or tartaric acid, vinegar injection (and acid baths—Ed.) All these means, however, are but palliatives; nothing but the termination of pregnancy, which is the cause, can cure the disease. The question as to the propriety of inducing artificial delivery, then, would naturally suggest itself, but it should not be attempted until symptoms of toxemia are sufficiently grave to threaten life. They are the only two indications for interference. To obviate congestion of the head, costiveness should be prevented by vinegar injections, aloes, jalap, etc.

The medical treatment of eclampsia during pregnancy, parturition, or childbed, is the same. The first indication is to diminish the reflex excitability, and thus ameliorate the paroxysms as much as possible, until rational or curative means can be instituted. One of the best, if not the best, is chloroform. The chloroform narcotism should be induced immediately upon the first appearance of the premonitory symptoms of the paroxysm, and kept up until quiet sleep is brought about. Dr. Braun states that sixteen cases of eclampsia occurring in succession, treated by chloroform and acid, complete recovery took place. To moderate the secondary congestion of the head, cold water poured from a height is the most effectual, sponging the skin with tepid vinegar brings about a very desirable diaphoresis, and should be frequently resorted to.

Dr. Braun is decidedly opposed to bleeding, unless in very plethoric patients, and then to quite a moderate extent. In this respect we decidedly disagree with him. Theory and experience both, we think, justify copious and prompt bleeding. Aside from the fact that we believe *congestio cerebri* is in many instances the main cause, we think, that if secondary, it calls for venesection when it does not exist, and that, if inflammation of the kidneys is the first and main pathological lesion, no means are calculated to relieve it than depletory. It must be acknowledged, however, that sixteen consecutive cases recovering cannot be regarded as accidental. Benzoic and vegetable acids, and much cold drinks, should be given constantly. During the paroxysm, no more restraint is necessary than merely to keep the patient from injuring herself by rolling off the bed, biting her tongue, etc. All agree that, if the os uteri is open sufficiently to allow the application of the forceps, it is best to extract at once, in cautious manner. If the os is not wide open, but dilating pretty rapidly, Dr. Braun would rupture the membranes, and allow the liquor amni to escape; if the convulsions do not cease, then the os should be further dilated with the fingers.

Dilation should be done thus under the influence of chloroform. If the head does not advance, and the paroxysms continue, the long forceps should be applied, and the head delivered.

On account of eclampsia alone, without any obstetrical disproportion of the pelvis, craniotomy should not be performed, nor should podalic version be performed, unless there is malposition, or obstetrical disproportion. Where there is no advance in labor, no dilatation of the os uteri, and it is desirable to deliver, the best way is to arouse uterine action, and dilate the os uteri.

The first may be done by passing a flexible catheter up into the uterine cavity, between the membranes and the walls of that organ, and allow it to remain long enough to induce contraction, and at the same time to keep the vagina in a state of distention, by inflating a gum elastic bag within its cavity. The latter expedient will dilate the os uteri pretty rapidly, and if allowed to remain two or three hours, will complete it sufficiently to allow the passage of the head. The passage of the elastic catheter between the membranes and the uterus for some distance is more efficient than rupture of the membranes. As before remarked, he deprecates forcible delivery by turning, thinking it never necessary, and often injurious. As they occur during pregnancy, before parturation has begun, nothing but extremity of danger from violent and frequent recurrence of the paroxysms would justify us in artificial deliverance. In such case catheterism and the use of the gum bag would be the best means. After delivery, the treatment of convulsions should be of that rational character above indicated. We would submit that in such cases, in addition to the acid diluent and diuretic treatment, bath and injections of vinegar, that cupping, fomentations to the loins, cold to the head, cathartics of croton oil, and venesection, should be used. We should have stated that Dr. Braun is an advocate, in addition to the use of chloroform, of large doses of opium, particularly when the chloroform abates in its controling effect. We have made the above curt abstract of Dr. B.'s little but excellent book, to give our readers some idea of its depth and thoroughness, hoping that it may incite to further examination of this very important subject, and we can assure them that they can best do so by attentively studying his essay.

Of Nature and Art in the Cure of Disease. By Sir John Forbes, M.D., D.C.L. (Oxon), F.R.S., Fellow of the Royal College of Physicians; Physician to the Queen's Household, etc. etc. From the second London edition. New York: Samuel S. & William Wood, 389 Broadway. 1858.

The perusal of Sir John's book is well calculated to lead to sober reflection as to the value of the various procedures of medication practiced at the present day. It is well occasionally thus to be brought to a stand while rushing with Young America speed through the various remedial measures being introduced, and inquire whether nature has not been injudiciously ignored in all our calculations as to the effects of remedies. This is plainly the opinion of Sir John Forbes. His is the position of conservatism, originating in the ripe experience and erudition of the writer, and, although we think he underrates the value of our medical art, and over-estimates the efforts of nature in the cure of disease, we believe that much very valuable advice is contained in the teachings of this book, and recommend it to the profession for mature consideration.

### NEW JOURNALS.

*Louisville Medical Gazette*, a semi-monthly, by Dr. L. F. Frazer; *The Semi-Monthly Medical News*, published in Louisville, by Professors S. M. Bemiss and J. W. Barron, of the University of Louisville; and *The Marysville Medical Gazette*, of California, published by Lorenzo Hubbard, M.D., and H. W. Tred, M.D.

We wish all the above enterprises abundant success, and shall be happy to welcome the journals to our table as exchanges every time they are issued.

The January and February numbers of the *London Lancet* have been received. As usual, the matter contained in them is select, varied and abundant. A library almost, as well as a chronicle of the times, it deserves more general patronage throughout the West particularly. A file of the *Lancet* for several years is a mirror of the profession in London, invaluable as a work of reference. The February number is embellished with an admirable likeness of Sir Benjamin Brodie.

*Braithwaite's Retrospect.*—Part 38 is at hand, with its encyclopedia contents, garnered from all parts of the world. How could we do without *Braithwaite's Retrospect*, or *Rankine's Abstract*, these times of the countless valuable, as well as valueless productions with which our periodicals teem? Much that ought never to be forgotten would pass into oblivion, were it not for the faithful collection and record made in these excellent abstracts of medical knowledge. Among the many great improvements made in medical teaching in the quarter of century just past, this is not the least, and we would urge medical men who desire to keep pace with the rapid march of the profession, to take both of them. They cost almost nothing compared to their value. A single number will give information that will pay more than two per cent. a month on the investment of a year's subscription, in cash, to say nothing of the reputation gained by the practice of their excellent teaching.

